# A Study to Assess the Association Between Sleep and Migraine

**DOI:** 10.7759/cureus.81453

**Published:** 2025-03-30

**Authors:** Amrit Bansod, Sushant Meshram, Sanjay Ramteke

**Affiliations:** 1 Neurology, Government Medical College and Hospital, Nagpur, IND; 2 Respiratory Medicine, Government Medical College and Hospital, Nagpur, IND

**Keywords:** headache, migraine disorder, pittsburgh sleep quality index (psqi), sleep disturbance, sleep quality

## Abstract

Introduction

Migraines and sleep disturbances affect the majority of people globally. The underlying pathophysiological basis for the role of sleep disturbances in the causation of migraine remains poorly understood due to the complexity of brain functions. As studies assessing the association between sleep disorders and migraine are scarce, the present study was conducted.

Materials and methods

This study was conducted after obtaining approval from the Institutional Ethics Committee. A total of 60 patients with migraine and 60 age- and gender-matched controls were included after obtaining voluntary, written, and informed consent, provided they met the inclusion and exclusion criteria. Demographic details and relevant histories were noted. Severity of migraine and sleep disturbances was assessed by self-reported questionnaires.

Results

In the present study, it was noted that both groups were similar in terms of demographic details and personal histories. The global Pittsburgh sleep quality index (PSQI) was significantly higher in cases as compared to controls. Strong positive correlations were noted between global PSQI and numerical pain rating (NPR), headache impact test (HIT-6), and migraine disability assessment (MIDAS) scores.

Conclusion

Patients with migraine have poor sleep quality and disturbed sleep. The sleep quality also worsens with an increase in the severity of the migraine. Therefore, it is recommended that sleep quality should be evaluated in the initial assessment, and treatment should be instituted accordingly.

## Introduction

Migraine is a common and widespread neurological disease. It is the second most common disability as per the Global Burden of Disease (GBD) study 2016 and affects approximately one billion people globally [1,2]. It has also been estimated that headaches in 75% of patients with migraines start before 35 years of age, with peak prevalence being in the age group of 35 to 39 years [2,3]. Migraine attacks are often accompanied by nausea and vomiting and sensitivity to light and sound. Migraine is known to severely affect the quality of life and lead to substantial disability. Multiple self-reported questionnaires have been developed for the assessment of the same.

Sleep is an important physiological phase of resting and convalescence and is associated with reduced motor activity and prepares the body for wakefulness [4]. Therefore, sleep disturbances may lead to various disorders. Around 45% of the global population is estimated to have some form of sleep disturbance [5].

The earliest published literature indicating the correlation between sleep and migraine dates back to the 19th century [6]. Subsequently, there has been a rising interest in exploring the association of migraine and sleep disorders to find out amenable ways for treatment. Over the years, there have been some studies documenting this relationship, but the underlying pathophysiological basis is still poorly understood due to the complexity of brain functions and due to the plethora of neurotransmitters involved. The present study was a cross-sectional, case-control study to evaluate the association of sleep and migraine using the standard self-reported questionnaires.

## Materials and methods

This cross-sectional, case-control study was conducted under the Department of Neurology at Government Medical College and Hospital, Nagpur, after obtaining approval from the Institutional Ethics Committee (4232/EC/Pharmac/GMC/NGP) on 30th May 2024. The study was conducted from 1st June 2024 to 30th November 2024. After obtaining voluntary, written, informed consent, 60 patients with migraine diagnosed as per the International Classification of Headache Disorders-3 (ICHD-3) criteria were included in the study [7]. Patients having severe illnesses requiring admission, diagnosed psychiatric or neurological illness (except migraine), and those refusing to give consent to participate in the study were excluded. Age- and gender-matched healthy volunteers having no personal or family history of migraine and having no history of diagnosis with any headache disorders were selected and included after taking voluntary, written, informed consent. Pregnant women and patients below the age of 18 years were excluded from both groups.

Demographic details were noted. For assessment of the sleep quality, the global Pittsburgh sleep quality index (PSQI) questionnaire was filled out [8]. This self-reported questionnaire assessed the subjective sleep quality, sleep latency, sleep duration, habitual sleep efficiency, sleep disturbances, use of sleep medication, and daytime dysfunction on a scale of zero to three. Accordingly, the total score for global PSQI was calculated. Additionally, the patients with migraine in the case group were also administered the numerical pain rating (NPR) questionnaire to assess the degree of pain, the six-item questionnaire for the headache impact test (HIT-6) for the assessment of the adverse impact of the migraine headaches, and the migraine disability assessment (MIDAS) questionnaire to assess the overall disability due to the migraine headaches (Appendices 1-5) [9,10,11].

Data was analyzed using the IBM SPSS Statistics for Windows, Version 28 (Released 2021; IBM Corp., Armonk, New York, United States). The categorical data was analyzed using the chi-square test (Fisher’s exact test for data with cell values less than five), and numerical data was analyzed using the student’s t-test. Correlation was assessed by the Pearson coefficient. A p-value of less than 0.05 was considered statistically significant.

## Results

The mean age of the cases was 37.97 ± 12.13 years, and the mean age of the controls was 36.70 ± 10.94 years. The age distribution was similar in the two groups; P-value: 0.549. There was a female preponderance in both groups (52 patients; 86.67% in cases and 50 patients; 83.33% in controls). The gender distribution was similar in the two groups; P-value: 0.609. The mean BMI of cases was 20.83 ± 3.51 kg/m², and of controls was 21.46 ± 3.30 kg/m², with a similar distribution in the two groups; P-value: 0.313.

The two groups were similar in terms of the presence of comorbidities of hypertension and diabetes mellitus; P-value: more than 0.05. The two groups were also similar in the presence of smoking and alcoholism; P-value: more than 0.05 (Table 1).

**Table 1:** Distribution of comorbidities and personal history The data for comorbidities and personal histories were presented as numbers and % for the two groups: cases and controls. A p-value of less than 0.05 was considered to be statistically significant. NA: not applicable. Fisher's exact test was performed as the cell value was less than 5. So, the chi-square value is not available for the parameter.

Parameter	Cases		Controls		Chi-square value	P-value
	N	%	N	%		
Hypertension	7	11.67%	11	18.33%	1.05	0.306
Diabetes mellitus	0	0%	4	6.67%	NA	0.119
Smoking	7	11.67%	6	10%	0.09	0.769
Alcoholism	9	15%	8	13.33%	0.07	0.793

When assessed for sleep quality, it was observed that the global PSQI score was significantly higher in the cases than in controls; P-value: less than 0.001 (Table 2).

**Table 2:** Distribution of global PSQI score The data for global PSQI was presented as mean and SD. A p-value of less than 0.05 was considered to be statistically significant. SD: standard deviation; PSQI: Pittsburgh sleep quality index *indicates statistically significant p-value

Parameter	Cases	Controls	t-value	P-value
	Mean ± SD	Mean ± SD		
Global PSQI for sleep	8.97 ± 4.08	4.92 ± 2.75	6.38	<0.001*

In sub-group analysis, it was observed that the global PSQI score showed a very strong positive significant correlation with the NPR scale and HIT-6 and a moderately strong positive correlation with the MIDAS score amongst the cases (Table 3).

**Table 3:** Correlation of global PSQI score with other scores in the case group The data for correlation of the global PSQI score with other scores was presented in terms of the Pearson coefficient. A p-value of less than 0.05 was considered to be statistically significant. BMI: body mass index; NPR: numerical pain rating; HIT-6: headache impact test-6; MIDAS: migraine disability assessment *indicates statistically significant p-value

Parameter	r (Pearson coefficient)	P-value
Age	0.11	0.406
BMI	0.09	0.516
NPR	0.72	<0.001*
HIT-6	0.71	<0.001*
MIDAS	0.67	<0.001*

## Discussion

In the present study, it was observed that the sleep quality was poor in the cases as compared to controls, as indicated by the higher global PSQI scores. Furthermore, the global PSQI score also correlated with the scores for severity of migraine and disability score, as indicated by the significant strong positive correlation.

A similar study was conducted by Krishna in 2021 [12]. A total of 70 migraine patients and 35 controls were included. The mean age of the cases was 33.48 ± 10.25 years, and of controls was 34.14 ± 9.41 years. There was a female preponderance in both groups (87.14% in cases and 82.86% in controls). The study reported that the global PSQI score was significantly higher in cases than in controls (8.94 ± 4.00 and 1.51 ± 1.14, respectively); P-value: less than 0.0001. The overall sleep quality was poor in cases as compared to controls when assessed by sleep diary. Insomnia was reported by 62.9% of the cases, while 45.7% of the cases reported taking more than 30 minutes to fall asleep, and 57.4% of the cases reported having less than six hours of sleep time. Similar results were also observed in another study by Lovati et al., wherein they reported worse sleep quality among patients with migraine as compared to patients without migraine [13]. In another study by Zhu et al., they reported that 61.6% of the patients with migraine had poor sleep quality (as indicated by the PSQI score of more than five) with increased latency period, daytime dysfunction, poor quality, and shorter duration [14]. Morgan et al. conducted their study in sub-Saharan Africa and reported worse PSQI scores amongst patients with migraine, along with deranged individual components of the PSQI score. They also reported reduced quality of life in patients with migraine in terms of poor physical and psychological health and poor social relationships [15]. These findings were similar to the present study.

Studies have documented the correlation between sleep disturbances and with frequency of headaches. In the study by Yeung et al. conducted on Chinese women, they reported that women with insomnia (insomnia severity index of more than seven) had a 3.2-fold increased risk of migraines, a 2.2-fold increased risk of recurrent headache, and a 2.3-fold increased risk of tension headache. They also reported that the association of insomnia with headaches increased with the frequency of headaches [16]. Lin et al. conducted their study in four groups according to the frequency of migraines and reported that the PSQI score was highest in the group with the highest frequency of migraine attacks; P-value: 0.006 [17]. In the present study, it was noted that the increase in the global PSQI score correlated well with the increase in the severity of migraine (NPR, HIT-6, and MIDAS scores).

Kim et al. conducted their study in Hong Kong and reported a bidirectional association between insomnia and migraine. They included 2695 patients and observed that the proportion of patients having migraine headaches was higher in patients with insomnia as compared to the patients without insomnia (12.9% and 4.4%, respectively); P-value: less than 0.001. They also reported that the insomnia severity index (ISI) score assessing the noticeability of sleep problems to others was significantly higher in cases with migraine as compared to those without migraine; P-value: 0.011 [18]. Similarly, the Nord-Trondelag Health Study (HUNT-2 and HUNT-3) trials have reported an increased risk of headaches in patients with insomnia. They also concluded that in initially headache-free patients, insomnia was associated with increased risk of headache after 11 years [19].

Sleep deprivation has been known to disturb the descending pain inhibitory control system, which may lead to increased pain sensations, particularly self-reported pain [20,21]. Sleep deprivation may interfere with normal functions and cause migraines at several levels. Lack of sleep causes changes in the concentration of neurotransmitters, like an increase in the concentration of adenosine, which is also a potent vasodilator and has a documented effect in causing migraines [22]. There may also be a disruption of the cyclical release of melatonin, an antinociceptive factor. Decreased levels of melatonin have also been documented during episodes of migraine [23,24]. Sleep deprivation may also lead to decreased cortical excitability, lower energy levels, and reduced neurogenesis. Dysfunction of the brainstem and hypothalamic structures is also speculated to be responsible for the development of migraine and sleep disorders, as these structures are often involved in the maintenance of the sleep-wake cycle [25]. A possible role of orexin-containing neurons has also been hypothesized, as they are involved in both sleep regulation and pain modulation [26]. Thus, sleep and migraine may have a bidirectional association that remains to be fully understood.

Limitations

The present study was a single-center study with a limited number of participants. Also, the study was limited by the outpatient department (OPD) attendance of the patients; therefore, the results may not be generalized.

## Conclusions

It can be effectively concluded from this study that poor sleep quality is a risk factor for migraine, though the bidirectional relationship needs to be explored. Furthermore, poor sleep quality also correlates with the severity of migraine and its impact on the quality of life. Therefore, it is recommended that the assessment of sleep disorders be included in the initial assessment protocol for migraine patients. In addition to the treatment of migraine, pharmacological interventions should be instituted for the treatment of sleep disorders at the earliest to prevent worsening of the condition and improve the quality of life of patients. Future research should consider a multicentric approach to increase external validity and better represent population diversity.

## Appendices

Appendix 1 

**Table 4:** Questionnaire format for collecting responses from patients BMI: body mass index; NPR: numerical pain rating; HIT-6: headache impact test-6; MIDAS: migraine disability assessment

Parameter	Response
Age (in years)	
Gender	Male/Female
BMI (in kg/m^2^)	
Hypertension	Present/Absent
Diabetes mellitus	Present/Absent
Smoking	Present/Absent
Alcoholism	Present/Absent
Global PSQI score (as calculated from the proforma)	
NPR score	From 0 (no pain) to 10 (worst possible pain)
HIT-6 score (as calculated from the proforma)	
MIDAS score (as calculated from the proforma)	

Appendix 2

The scores for the various components were calculated as per the standard questionnaire referenced in the Materials and Methods section. The questionnaire format for global PSQI is outlined in Appendix 2.

**Table 5:** Questionnaire format for collection of responses for the assessment of global PSQI score Numbers in brackets indicate score. PSQI: Pittsburgh sleep quality index

Question	Response			
1. During the past month, what time have you usually gone to bed at night?				
2. During the past month, how long (in minutes) has it usually taken you to fall asleep each night?	Less than 15 minutes (0)	16 to 30 minutes (1)	31 to 60 minutes (2)	More than 60 minutes (3)
3. During the past month, what time have you usually gotten up in the morning?				
4. During the past month, how many hours of actual sleep did you get at night?	More than 7 hours (0)	6 to 7 hours (1)	5 to 6 hours (2)	Less than 5 hours (3)
5. During the past month, how often have you had trouble sleeping because you…				
a. Cannot get to sleep within 30 minutes	Not during the past month (0)	Less than once a week (1)	Once or twice a week (2)	Three or more times a week (3)
b. Wake up in the middle of the night or early morning	Not during the past month (0)	Less than once a week (1)	Once or twice a week (2)	Three or more times a week (3)
c. Have to get up to use the bathroom	Not during the past month (0)	Less than once a week (1)	Once or twice a week (2)	Three or more times a week (3)
d. Cannot breathe comfortably	Not during the past month (0)	Less than once a week (1)	Once or twice a week (2)	Three or more times a week (3)
e. Cough or snore loudly	Not during the past month (0)	Less than once a week (1)	Once or twice a week (2)	Three or more times a week (3)
f. Feel too cold	Not during the past month (0)	Less than once a week (1)	Once or twice a week (2)	Three or more times a week (3)
g. Feel too hot	Not during the past month (0)	Less than once a week (1)	Once or twice a week (2)	Three or more times a week (3)
h. Have bad dreams	Not during the past month (0)	Less than once a week (1)	Once or twice a week (2)	Three or more times a week (3)
i. Have pain	Not during the past month (0)	Less than once a week (1)	Once or twice a week (2)	Three or more times a week (3)
j. Other reason(s), please describe:	Not during the past month (0)	Less than once a week (1)	Once or twice a week (2)	Three or more times a week (3)
6. During the past month, how often have you taken medicine to help you sleep (prescribed or “over the counter”)?	Not during the past month (0)	Less than once a week (1)	Once or twice a week (2)	Three or more times a week (3)
7. During the past month, how often have you had trouble staying awake while driving, eating meals, or engaging in social activity?	Not during the past month (0)	Less than once a week (1)	Once or twice a week (2)	Three or more times a week (3)
8. During the past month, how much of a problem has it been for you to keep up enough enthusiasm to get things done?	No problem at all (0)	Only a very slight problem (1)	Somewhat of a problem (2)	Very big problem (3)
9. During the past month, how would you rate your sleep quality overall?	Very good (0)	Fairly good (1)	Fairly bad (2)	Very bad (3)
10. Do you have a bed partner or roommate?	No bed partner or roommate	Partner/roommate in other room	Partner in the same room but not the same bed	Partner in the same bed
If you have a roommate or bed partner, ask him/her how often in the past month you have had:	Not during the past month	Less than once a week	Once or twice a week	Three or more times a week
a. Loud snoring				
b. Long pauses between breaths while asleep				
c. Legs twitching or jerking while you sleep				
d. Episodes of disorientation or confusion during sleep				
e. Other restlessness while you sleep, please describe:				

Appendix 3

The scores from questions in Appendix 2 are summed up for the component scores as per Appendix 3. The individual component scores are summed up for the global PSQI score.

**Table 6:** Calculation of component scores of global PSQI from the individual questions PSQI: Pittsburgh sleep quality index

Component	Parameter	Question	Remarks
Component 1	Subjective sleep quality	Q9	Final score is same as response
Component 2	Sleep latency	Q2 and Q5a	Final score is: If sum is 0: Score 0; If sum is 1-2: Score 1; If sum is 3-4: Score 2; If sum is 5-6: Score 3
Component 3	Sleep duration	Q4	Final score is same as response
Component 4	Sleep efficiency	Q1, Q3 and Q4	Sleep efficiency= (number of hours slept/number of hours in bed) × 100. Final score is: For more than 85%: Score 0; 75-84%: Score 1; 65-74%: Score 2; Less than 65%: Score 3
Component 5	Sleep disturbance	Q5b to Q5j	Final score is: If sum is 0: Score 0; If sum is 1-9: Score 1; If sum is 10-18: Score 2; If sum is 19-27: Score 3
Component 6	Use of sleep medication	Q6	Final score is same as response
Component 7	Daytime dysfunction	Q7 and Q8	Final score is: If sum is 0: Score 0; If sum is 1-2: Score 1; If sum is 3-4: Score 2; If sum is 5-6: Score 3

Appendix 4

The questionnaire for HIT-6 is outlined in Appendix 4. The final score is calculated as the sum of all responses.

**Table 7:** Questionnaire format for collection of responses for the assessment of HIT-6 score Numbers in brackets indicate score. HIT-6: headache impact test-6

Question	Never (6)	Rarely (8)	Sometimes (10)	Very often (11)	Always (13)
When you have headaches, how often is the pain severe?					
How often do headaches limit your ability to do usual daily activities, including household work, work, school, or social activities?					
When you have a headache, how often do you wish you could lie down?					
In the past four weeks, how often have you felt too tired to do work or daily activities because of your headaches?					
In the past four weeks, how often have you felt fed up or irritated because of your headaches?					
In the past four weeks, how often did headaches limit your ability to concentrate on work or daily activities?					
Total Score (sum of all responses)					

Appendix 5

The questionnaire for MIDAS is outlined in Appendix 5. The final score is calculated as the sum of all responses.

**Table 8:** Questionnaire format for collection of responses for the assessment of the MIDAS score MIDAS: migraine disability assessment

Question	Frequency in the past 3 months
On how many days in the last 3 months did you miss work or school because of your headaches?	
How many days in the last 3 months was your productivity at work or school reduced by half or more because of your headaches?	
On how many days in the last 3 months did you not do household work (such as housework, home repairs and maintenance, shopping, caring for children and relatives) because of your headaches?	
How many days in the last 3 months was your productivity in household work reduced by half or more because of your headaches?	
On how many days in the last 3 months did you miss family, social, or leisure activities because of your headaches?	
Total Score (sum of all responses)	
